# Intracellular Group A *Streptococcus* Induces Golgi Fragmentation To Impair Host Defenses through Streptolysin O and NAD-Glycohydrolase

**DOI:** 10.1128/mBio.01974-20

**Published:** 2021-02-09

**Authors:** Takashi Nozawa, Junpei Iibushi, Hirotaka Toh, Atsuko Minowa-Nozawa, Kazunori Murase, Chihiro Aikawa, Ichiro Nakagawa

**Affiliations:** a Department of Microbiology, Graduate School of Medicine, Kyoto University, Kyoto, Japan; College of Veterinary Medicine, Cornell University

**Keywords:** Golgi apparatus, group A *Streptococcus*, IL-8, NAD-glycohydrolase, streptolysin O

## Abstract

Group A *Streptococcus* (GAS; Streptococcus pyogenes) is a major human pathogen that causes streptococcal pharyngitis, skin and soft tissue infections, and life-threatening conditions such as streptococcal toxic-shock syndrome. During infection, GAS not only invades diverse host cells but also injects effector proteins such as NAD-glycohydrolase (Nga) into the host cells through a streptolysin O (SLO)-dependent mechanism without invading the cells; Nga and SLO are two major virulence factors that are associated with increased bacterial virulence. Here, we have shown that the invading GAS induces fragmentation of the Golgi complex and inhibits anterograde transport in the infected host cells through the secreted toxins SLO and Nga. GAS infection-induced Golgi fragmentation required both bacterial invasion and SLO-mediated Nga translocation into the host cytosol. The cellular Golgi network is critical for the sorting of surface molecules and is thus essential for the integrity of the epithelial barrier and for the immune response of macrophages to pathogens. In epithelial cells, inhibition of anterograde trafficking by invading GAS and Nga resulted in the redistribution of E-cadherin to the cytosol and an increase in bacterial translocation across the epithelial barrier. Moreover, in macrophages, interleukin-8 secretion in response to GAS infection was found to be suppressed by intracellular GAS and Nga. Our findings reveal a previously undescribed bacterial invasion-dependent function of Nga as well as a previously unrecognized GAS-host interaction that is associated with GAS pathogenesis.

## Abstract (precis)

Two prominent virulence factors of group A *Streptococcus* (GAS), streptolysin O (SLO) and NAD-glycohydrolase (Nga), are linked to enhanced pathogenicity of the prevalent GAS strains. Recent advances show that SLO and Nga are important for intracellular survival of GAS in epithelial cells and macrophages.

## INTRODUCTION

Group A *Streptococcus* (GAS; Streptococcus pyogenes) is a human-specific pathogen responsible for diverse diseases, ranging from pharyngitis and impetigo to life-threatening conditions such as necrotizing fasciitis and streptococcal toxic-shock syndrome (STSS) in which mortality rates are 30% to 70%, even with immediate antibiotic therapy and intensive care (1). Therefore, GAS species are commonly referred to as “killer bacteria” or “flesh-eating bacteria,” and the ability of GAS to spread rapidly at the infection site and disseminate systemically indicates that the pathogen possesses robust mechanisms to resist the human innate immune response.

The initial sites of GAS infection are the pharyngeal epithelia and the keratinocytes, and the pathogen invades deeper tissues through the paracellular pathway by degrading the junctional proteins. Although GAS is commonly regarded as an extracellular pathogen, it can invade epithelial cells, endothelial cells, and macrophages, and this cellular invasion has been reported to be associated with GAS pathogenesis (2–5). However, several GAS strains, except for certain isolates, are degraded through the endosomal pathway or autophagy and cannot survive for long periods inside the epithelial cells (6–11), and the importance of GAS invasion into host cells remains incompletely elucidated.

Recent transcriptome evidence has revealed that highly virulent GAS strains exhibit enhanced expression of two toxins, streptolysin O (SLO) and NAD-glycohydrolase (Nga) (12–14), which emphasizes a role for these toxins in GAS pathogenesis. SLO is a member of the family of cholesterol-dependent cytolysins that bind to the cholesterol-containing membranes, oligomerize, and insert into the lipid bilayer to form pores (15–17). SLO not only induces the necrosis of neutrophils through pore formation (17, 18) but also translocates the effector protein Nga into the host cytosol in a pore formation-independent manner, thereby promoting intracellular survival in macrophages and epithelial cells (19–21). Nga hydrolyzes NAD into nicotinamide and ADP-ribose and thus depletes intracellular NAD pools and causes ATP depletion in cells. Accordingly, Nga has been reported to inhibit the acidification of phagosomes or autolysosomes potentially through ATP depletion in macrophages and keratinocytes (9, 22). Moreover, Nga inhibits the canonical autophagy pathway to promote bacterial survival in epithelial cells (23) and extracellularly inhibits interleukin-1 beta (IL-1β) production (21). Nicotinamide potently inhibits the secretion of proinflammatory cytokines from monocytes (24). These lines of evidence have established that SLO and Nga enable GAS to persist within host cells and to modulate immune responses, and these effects are considered to be exerted by the Nga activity itself.

Pathogenic bacteria evade host defenses by subverting the host signaling pathways through several distinct and sophisticated mechanisms (25, 26). For example, Legionella pneumophila, Chlamydia trachomatis, and Burkholderia thailandensis secrete the SET domain-containing proteins that methylate histones to alter the chromatin landscape of the host cell (27–29) and thus promote the intracellular proliferation of bacteria. Salmonella enterica serovar Typhimurium, *Legionella* spp., and *Brucella* spp. modulate host membrane dynamics to allow the bacteria to form replication-permissive vacuoles (25). Enteropathogenic Escherichia coli and Shigella flexneri target the Golgi network, the endoplasmic reticulum (ER), and the eukaryotic secretory pathway to suppress host defenses (30, 31). Here, to uncover the previously unrecognized GAS-host interactions, we examined the organelle morphology in host cells during GAS infection, which revealed that GAS infection triggers the fragmentation of the Golgi complex. We determined that SLO and Nga were responsible for this effect and, furthermore, that both SLO-mediated Nga translocation and bacterial invasion into host cells were required for disruption of the Golgi network. Inhibition of the Golgi network resulted in the loss of not only epithelial integrity but also IL-8 secretion by macrophages in response to GAS infection.

## RESULTS

### Golgi apparatus is fragmented during GAS infection.

**FIG 1:**
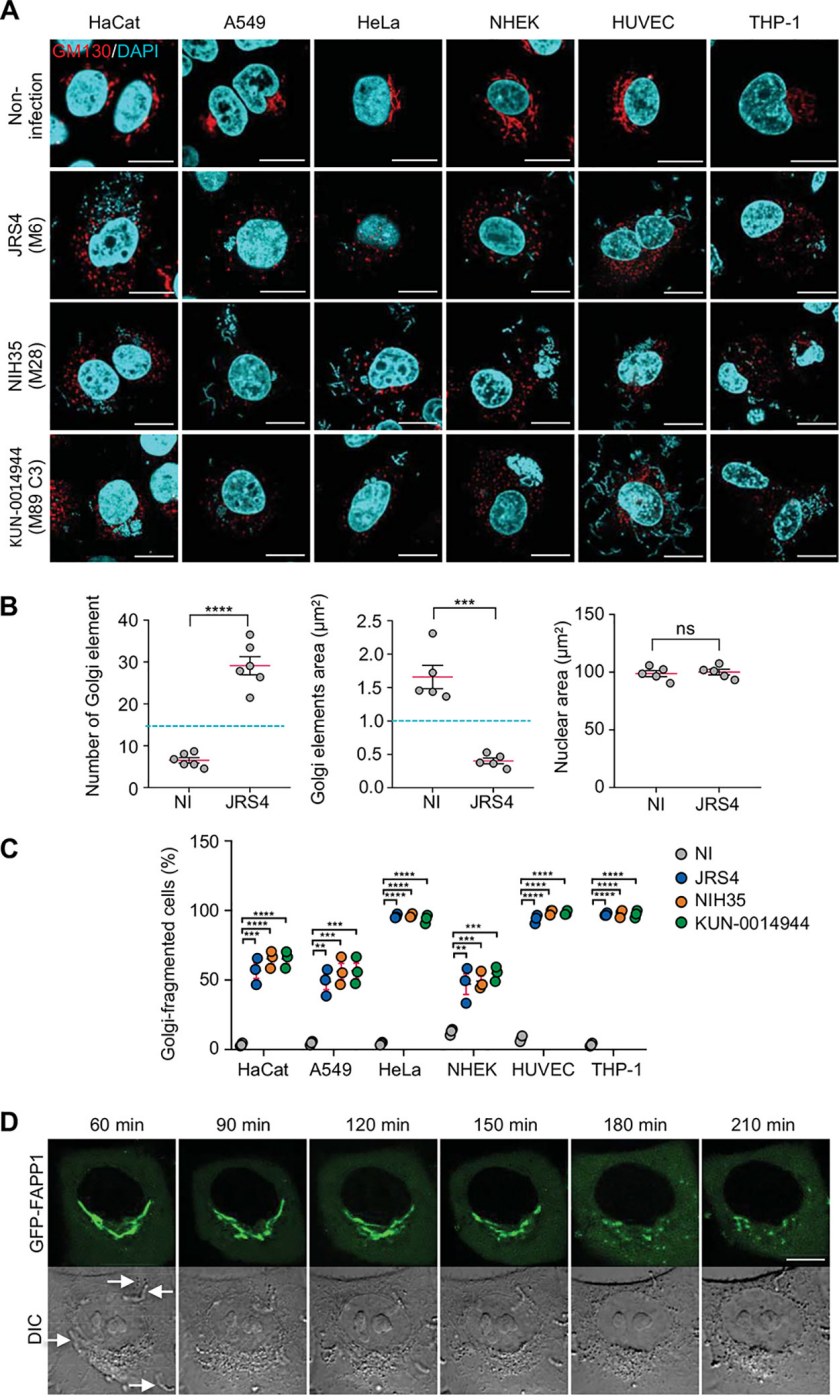
GAS induces Golgi fragmentation in infected host cells. (A and B) Golgi structure during GAS infection. Cells were infected with indicated GAS strains for 4 h, fixed, and immunostained for the Golgi marker GM130 (red). Cellular and bacterial DNA was stained with DAPI (cyan). Representative confocal images (A) and quantification (B) of the Golgi and nucleus signals. (C) Quantification of the Golgi-fragmented cells that showed >15 Golgi elements that were <1 μm^2^. (D) EmGFP-FAPP1-expressing HeLa cells were infected with GAS JRS4. Confocal images were captured at the indicated times after infection. The arrows indicate invading GAS. Scale bars, 10 μm. Data in panels B and C represent individual values (dots) (*n* > 20 cells per condition) and the means (magenta lines) ± SEMs from independent experiments. *P* values calculated by two-tailed Student’s *t* test. **, *P* < 0.01, ***, *P* < 0.001, ****, *P* < 0.0001; ns, not significant.

Because intracellular signaling and vesicular trafficking are closely associated with organelles, disruptions of host functions frequently result in alterations in the organelle morphology. Therefore, we examined the mechanism by which GAS infection affects intracellular vesicular or signaling networks. We infected HeLa cells with the GAS JRS4 strain, an M6 strain that efficiently invades host cells. The infected cells were immunostained for a number of organelle marker proteins to compare the morphology of the mitochondria, ER, *cis*-Golgi network, and *trans*-Golgi network before and after infection. Notably, the infection produced overt morphological changes in the mitochondria and *cis*/*trans*-Golgi network (see Fig. S1 in the supplemental material). During GAS infection, the normal tubular network of the mitochondria was fragmented into short rods or spheres, and the typical ribbon-like structure of the Golgi complex was also fragmented into punctate structures and dispersed throughout the cytoplasm (Fig. S1). We have previously reported that GAS invasion triggers apoptotic signaling, which causes mitochondrial fragmentation (32). Thus, in the present study, we examined Golgi fragmentation during GAS infection in more detail. To test whether this infection-induced Golgi fragmentation was observed in different cell types, we used GAS JRS4 to infect lung epithelial cells (A549 cells), human keratinocytes (HaCat cells), primary dermal keratinocytes (normal human epidermal keratinocytes [NHEKs]), human umbilical vein endothelial cells (HUVECs), and a human monocyte leukemia cell line (THP-1). JRS4 infection fragmented the Golgi structures, which then appeared dispersed throughout the cytoplasm in all types of cells tested (Fig. 1A). We also infected these cells with two other GAS strains, NIH35, a serotype M28 invasive strain isolated from an STSS patient, and KUN-0014944, an epidemic serotype M89 clade-3 strain. Both GAS strains also clearly caused Golgi fragmentation in all the cells examined (Fig. 1A).

10.1128/mBio.01974-20.1 FIG S1 GAS infection induces the fragmentation of the Golgi apparatus and mitochondria. HeLa cells were infected with GAS JRS4 for 4 h, fixed, and immunostained with the indicated antibodies. Download FIG S1, PDF file, 0.1 MB. Copyright © 2021 Nozawa et al. 2021 Nozawa et al. This content is distributed under the terms of the Creative Commons Attribution 4.0 International license.

To quantify the aforementioned phenotype, we measured the number and area of Golgi elements that were positive for the Golgi marker GM130. We found that the JRS4-infected cells showed >20 Golgi elements, and the area of each Golgi element was 0.3 to 0.6 μm^2^ (Fig. 1B). We next defined cells containing >15 Golgi elements featuring an area of 1.0 μm^2^ as the Golgi-fragmented cells, and we found that the Golgi fragmentation efficiencies were similar among the GAS strains (Fig. 1C); >90% of the infected HeLa cells, HUVECs, and THP-1 cells exhibited Golgi fragmentation at 4 h postinfection, whereas 40% to 60% of the HaCat, A549, and NHEK cells showed Golgi fragmentation (Fig. 1C).

To examine the time course of the changes in the Golgi apparatus structure during GAS infection, we expressed emerald green fluorescent protein (EmGFP)-tagged FAPP1 (a Golgi apparatus-resident protein) in cells and performed time-lapse imaging during the infection. In live-cell microscopy, the Golgi fragmentation process was detected at 2 to 3 h postinfection (Fig. 1D).

Golgi fragmentation has been reported in apoptotic cells (33). Thus, to examine whether the infection-induced fragmentation here was caused by apoptotic signaling, we inhibited apoptotic signaling by overexpressing the antiapoptotic protein Bcl-2 (34); ∼90% of the Bcl-2-expressing GAS-infected cells exhibited Golgi fragmentation (see Fig. S2A). Moreover, fragmentation was also not inhibited when infected cells were treated with the pan-caspase inhibitor Z-VAD-FMK (an inflammatory-caspase inhibitor) (Fig. S2B). These results suggest that apoptotic signaling may not be involved in the Golgi fragmentation that occurs during GAS infection. Collectively, our findings suggest that GAS infection triggers the fragmentation of the Golgi apparatus in various types of human cells.

10.1128/mBio.01974-20.2 FIG S2 Inhibition of apoptotic signal does not suppress Golgi fragmentation during GAS infection. (A) HeLa Bcl-2-expressing cells were infected with GAS JRS4 for 4 h and immunostained for GM130. (B) HeLa cells were treated with a pan-caspase inhibitor, Z-VAD-FMK (50 μM), infected with GAS JRS4 for 4 h, and immunostained for GM130. The percentages of cells showing Golgi fragmentation were quantified. (C) HeLa cells were infected with JRS4 at an MOI 10 for 4 h and immunostained for SLO and GM130. Arrows indicate cytosolic GAS. Data in panels A and B represent individual values (dots) (*n* > 20 cells per condition) and the means (magenta lines) ± SEMs from independent experiments. Scale bars, 10 μm. Download FIG S2, PDF file, 0.1 MB. Copyright © 2021 Nozawa et al. 2021 Nozawa et al. This content is distributed under the terms of the Creative Commons Attribution 4.0 International license.

To examine the possibility that a high burden of bacteria can cause this phenotype, we infected cells at a lower multiplicity of infection (MOI) and stained cytosolic GAS with the anti-SLO antibody. We previously showed that cytosolic GAS can be stained with this antibody (35). We observed that cells with only one or a few cytosolic GASs also showed Golgi fragmentation (Fig. S2C), suggesting that Golgi fragmentation can be induced sufficiently by a small number of GASs invading the host cytosol.

### SLO and Nga are critical for GAS-induced Golgi fragmentation.

**FIG 2:**
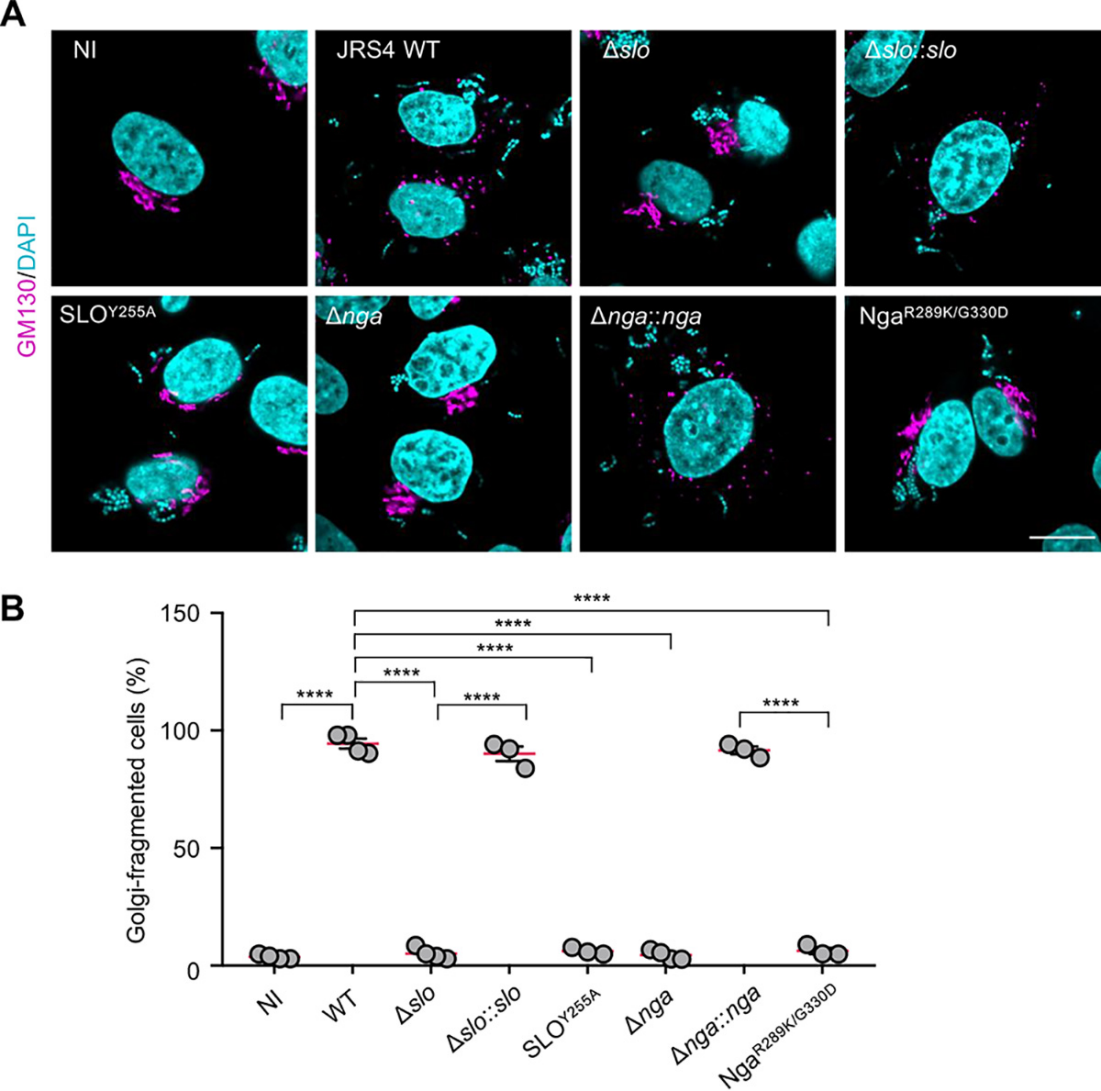
Golgi fragmentation during GAS infection requires SLO and Nga. (A and B) HeLa cells were infected with the indicated GAS strains for 4 h, fixed, and immunostained for GM130 (magenta). Representative confocal images (A) and quantification (B) of the cells with fragmented Golgi during infection. Scale bar, 10 μm. Data in panel B represent individual values (dots) (*n* > 20 cells per condition) and the means (magenta lines) ± SEMs from independent experiments. *P* values calculated by two-tailed Student’s *t* test. ****, *P* < 0.0001.

Pathogenic bacteria inject virulence effector proteins into the host cells to modulate host cellular processes. GAS can deliver effector proteins across the host plasma membrane or the endosomal membrane to modulate host signaling by cytolysin-mediated translocation (CMT) that uses pore-forming cytolysin SLO. Therefore, to examine whether SLO functions in the infection-induced Golgi fragmentation described above, we infected the HeLa cells with an SLO-deficient mutant (Δ*slo*). Infection with JRS4 Δ*slo* did not induce Golgi fragmentation, whereas complementation with *slo* completely rescued the phenotype (Fig. 2A and B). Moreover, JRS4 SLO^Y255A^, a mutant that lacks the pore-forming activity of SLO (20), failed to induce Golgi fragmentation (Fig. 2A and B), which indicates that GAS-induced Golgi fragmentation involves the pore-forming activity of SLO during infection.

SLO is expressed from an operon that also encodes Nga. SLO is necessary for Nga translocation into the host cells (19). To ascertain whether Nga was also required for GAS-induced Golgi fragmentation or whether SLO pore-forming activity directly triggered the fragmentation, we examined the Golgi morphology in the cells infected with JRS4 Δ*nga* and Δ*nga*::*nga* (*nga*-complemented strain). The Golgi structures in the Δ*nga* mutant-infected cells but not the Δ*nga*::*nga* complement-infected cells were found to be compact, and quantification of the Golgi signals indicated that *nga* was critical for GAS-induced Golgi fragmentation (Fig. 2A and B). We also confirmed that SLO and Nga were crucial for the Golgi fragmentation induced by strain NIH35 (see Fig. S3A and B).

10.1128/mBio.01974-20.3 FIG S3 GAS NIH35 strain induces Golgi fragmentation in an SLO- and Nga-dependent manner. (A) HeLa cells were infected with NIH35 strains for 4 h and immunostained for GM130. Scale bar, 10 μm. (B) The percentages of cells showing Golgi fragmentation were quantified. Data represents individual values (dots) (*n* > 20 cells per condition) and the means (magenta lines) ± SEMs from independent experiments. Download FIG S3, PDF file, 0.1 MB. Copyright © 2021 Nozawa et al. 2021 Nozawa et al. This content is distributed under the terms of the Creative Commons Attribution 4.0 International license.

To test whether the NADase activity of Nga was responsible for the Golgi fragmentation, we infected cells with the strain JRS4 Nga^R289K/G330D^; the mutations in Nga in the present study abolished the NADase activity of the effector (36). Although JRS4 Nga^R289K/G330D^ can invade the host cytosol (23), we observed no alteration of the Golgi structure during JRS4 Nga^R289K/G330D^ infection (Fig. 2A and B). Taken together, these data indicate that SLO pore-forming activity and Nga NADase activity are required for GAS-induced Golgi fragmentation.

GAS also harbors cytolytic toxin streptolysin S (SLS), and *sagA* is a structural gene for SLS (37). To examine if the Golgi fragmentation occurs independently or synergistically with SLS, we infected cells with the SLS-deficient mutant of GAS (Δ*sagA*). Infection with JRS4 Δ*sagA* induced Golgi fragmentation to the same extent as WT JRS4 (see Fig. S4A and B). Therefore, it is suggested that the Golgi fragmentation during GAS infection occurs independently of SLS.

10.1128/mBio.01974-20.4 FIG S4 Involvement of SLS in GAS-induced Golgi fragmentation. (A) HeLa cells were infected with JRS4 strains for 4 h and immunostained for GM130. Scale bars, 10 μm. (B) The percentages of cells showing Golgi fragmentation were quantified. Data represents individual values (dots) (*n* > 20 cells per condition) and the means (magenta lines) ± SEMs from independent experiments. Download FIG S4, PDF file, 0.1 MB. Copyright © 2021 Nozawa et al. 2021 Nozawa et al. This content is distributed under the terms of the Creative Commons Attribution 4.0 International license.

### GAS invasion is required for Nga-mediated Golgi fragmentation during infection.

**FIG 3:**
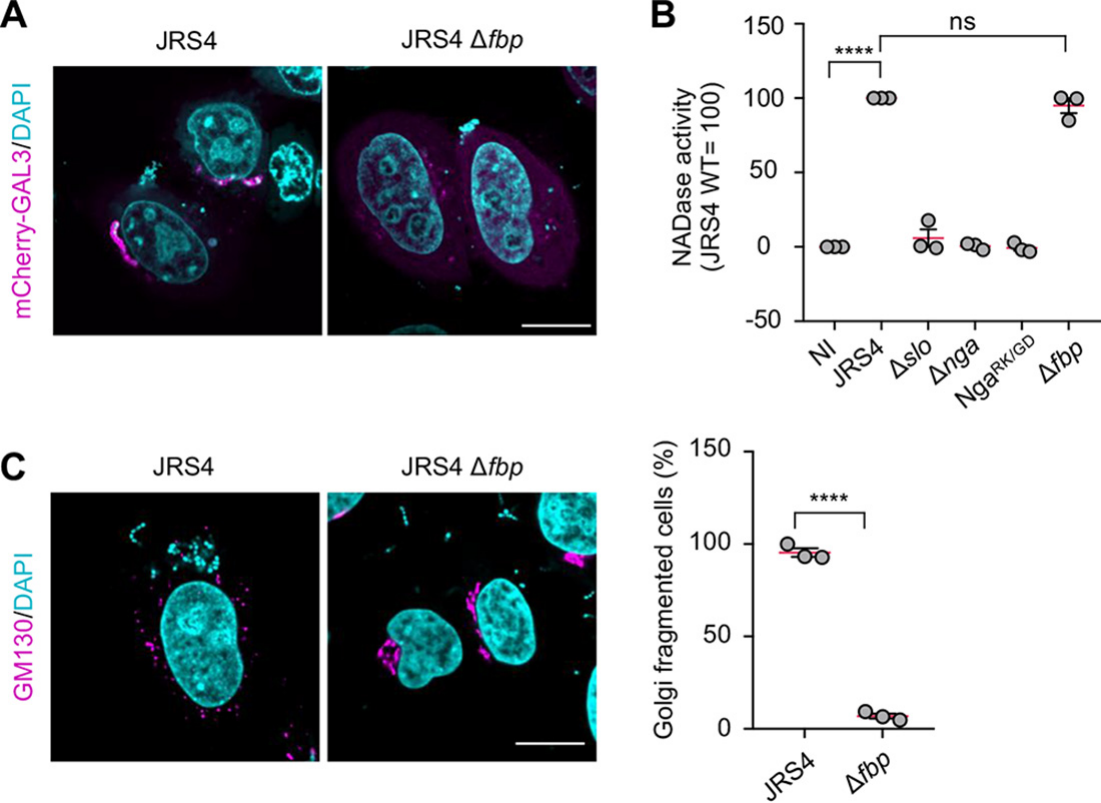
GAS invasion is necessary for Nga-mediated Golgi fragmentation. (A) HeLa cells transiently expressing mCherry-galectin-3 (GAL3) were infected with JRS4 wild-type or the Δ*fbp* mutant for 4 h. mCherry-GAL3-positive Δ*fbp* mutants were not observed. (B) NADase activity was assessed by measuring NAD consumption in the cytosolic fractions of infected cells. (C) HeLa cells were infected with the indicated GAS strains for 4 h, fixed, and immunostained for GM130 (magenta). Scale bars, 10 μm. Data in panels B and C represent individual values (dots) (*n* > 20 cells per condition) and the means (magenta lines) ± SEMs from independent experiments. *P* values calculated by two-tailed Student’s *t* test. ****, *P* < 0.0001; ns, not significant.

We next examined whether Golgi fragmentation was triggered by extracellular GAS. Because GAS JRS4 requires fibronectin-binding protein (FBP) to invade host cells (38), we constructed the strain JRS4 Δ*fbp* and infected the HeLa cells with this mutant; moreover, to monitor GAS invasion into the host cytosol, we expressed mCherry-galectin-3, which serves as a marker of damaged vacuoles when invasive pathogens escape into the cytosol (39). Our results confirmed that JRS4 Δ*fbp* was unable to invade HeLa cells (Fig. 3A). Next, we tested whether JRS4 Δ*fbp* translocates Nga into the cytosol by analyzing the NADase activity, assessed based on NAD consumption, in the cytosol of the HeLa cells after infection. As hypothesized, after infection with the JRS4 wild-type and Δ*fbp* strains, we measured comparable levels of NADase activity in the cytosol of the HeLa cells, which demonstrated that Nga was translocated across the host cell membrane even during infection with JRS4 Δ*fbp* (Fig. 3B). Unexpectedly, however, JRS4 Δ*fbp* failed to induce Golgi fragmentation (Fig. 3C). These results suggest that Golgi fragmentation requires not only SLO and Nga but also GAS invasion into the host cells.

To examine the possibility that bacterial adherence or internalization of host cells might be required for the Nga-mediated Golgi fragmentation, we treated the cells with cytochalasin D (cytD) to inhibit GAS invasion; cytD treatment does not affect SLO-mediated translocation of Nga (21). Notably, cytD treatment markedly suppressed Golgi fragmentation during JRS4 infection (see Fig. S5). Together, these results show that both GAS invasion and SLO-mediated injection of Nga into the host cells are critical for GAS-induced Golgi fragmentation.

10.1128/mBio.01974-20.5 FIG S5 GAS invasion is necessary for Golgi fragmentation. HeLa cells treated with cytD (5 μg/ml) were infected with GAS JRS4 for 3 h. Cells were immunostained for GM130. Scale bar, 10 μm. The percentages of cells showing Golgi fragmentation were quantified. Data represent individual values (dots) (*n* > 20 cells per condition) and the means (magenta lines) ± SEMs from independent experiments. Download FIG S5, PDF file, 0.1 MB. Copyright © 2021 Nozawa et al. 2021 Nozawa et al. This content is distributed under the terms of the Creative Commons Attribution 4.0 International license.

### GAS impairs the anterograde transport pathway.

**FIG 4:**
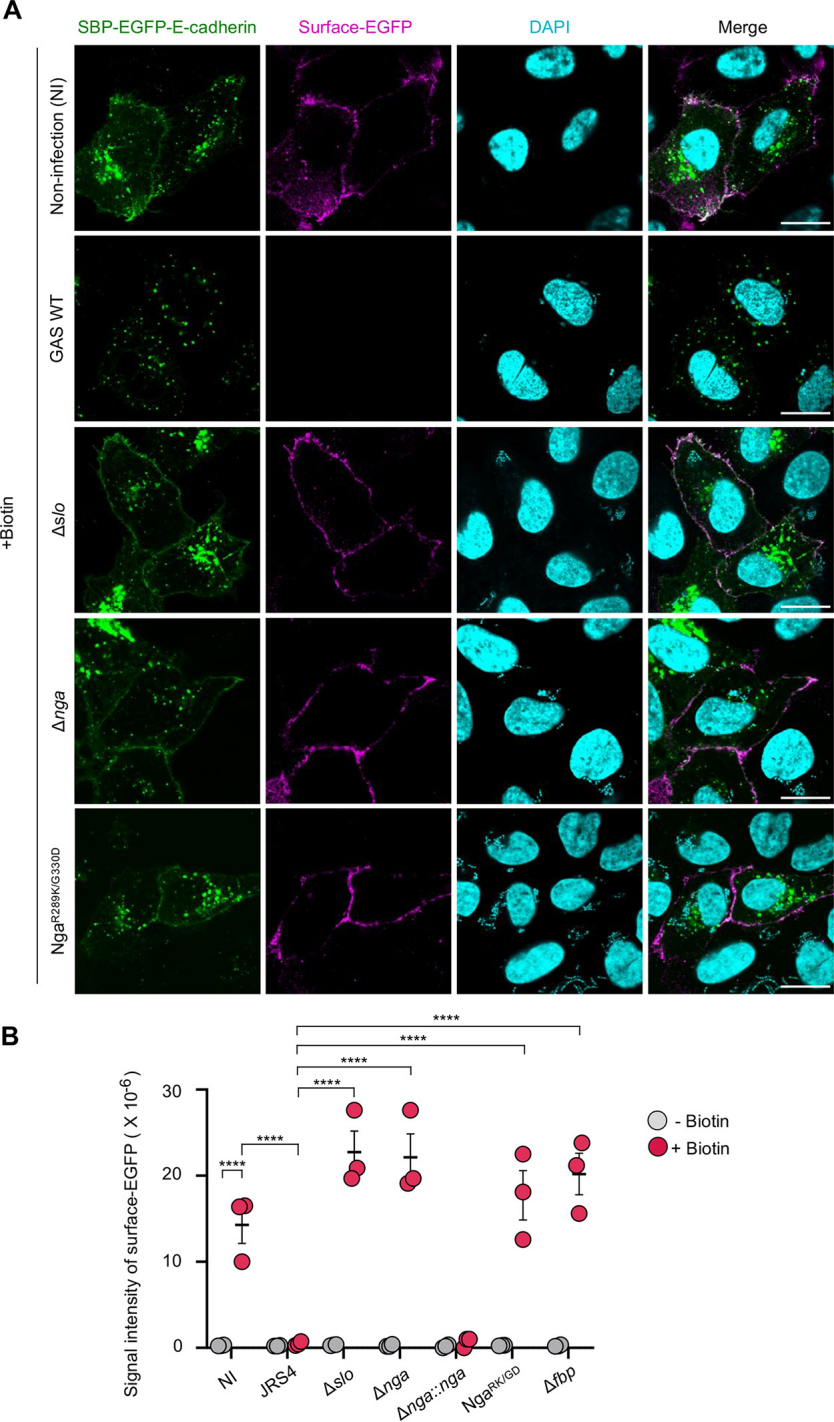
GAS inhibits anterograde transport through SLO and Nga. (A) Anterograde trafficking was inhibited in GAS-infected cells. HeLa cells expressing streptavidin-KDEL and SBP-EGFP-E-cadherin were infected with GAS strains. Cells were infected with GAS for 2 h and then incubated for 1 h with biotin to observe the traffic of SBP-EGFP-E-cadherin to the plasma membrane. Cells were then fixed, and surface E-cadherin was detected with an anti-GFP (magenta) prior to cell permeabilization. Cellular and bacterial DNA was stained with DAPI (cyan). Scale bars, 10 μm. (B) Quantification of surface E-cadherin using anti-GFP immunostaining. Average intensity of regions of interest corresponding to transfected cells was measured. Data represent individual values (dots) (*n* > 20 cells per condition) and the means (black lines) ± SEMs from independent experiments. *P* values calculated by two-tailed Student’s *t* test. ****, *P* < 0.0001.

The Golgi apparatus functions in mediating protein and lipid modifications, transport, and sorting. To assess whether the post-Golgi secretion pathway was inhibited by GAS, we examined anterograde transport by using the retention using selective hooks (RUSH) system (40). In our assay, E-cadherin was fused to a streptavidin-binding peptide (SBP) and enhanced green fluorescent protein (EGFP) and coexpressed with streptavidin-KDEL, which localizes in the ER. Under normal conditions, interaction of SBP-EGFP-E-cadherin with streptavidin-KDEL in the ER prevented the transport of the fusion protein to the plasma membrane (see Fig. S6). However, after the addition of biotin, which competes with the SBP tag for streptavidin binding, SBP-EGFP-E-cadherin was released from the ER and transported to the plasma membrane through the Golgi complex in the noninfected cells, and the E-cadherin that is normally trafficked to the plasma membrane was detected by immunostaining for EGFP without membrane permeabilization (Fig. 4A). In contrast, in the JRS4-infected cells, SBP-EGFP-E-cadherin exhibited punctate localization and the surface EGFP signal was rarely detected (Fig. 4A). Quantification of the surface EGFP signal revealed that anterograde trafficking of E-cadherin was abolished in the JRS4-infected cells (Fig. 4B). We also infected cells with Δ*slo*, Δ*nga*, Δ*nga*::*nga*, and Nga^R289K/G330D^ mutants, and found that while Δ*slo*, Δ*nga*, and Nga^R289K/G330D^ mutants did not affect anterograde trafficking, Δ*nga*::*nga* mutant infection inhibited the trafficking as effectively as JRS4 wild-type infection (Fig. 4A and B). Collectively, these results suggest that Golgi fragmentation is caused by the invading GAS, and the effector Nga results in a defect in the host-cell anterograde trafficking.

10.1128/mBio.01974-20.6 FIG S6 Confocal images of the control condition in the RUSH assay. HeLa cells expressing streptavidin-KDEL and SBP-EGFP-E-cadherin were infected with GAS strains. Cells were then fixed, and the surface E-cadherin was detected with an anti-GFP antibody (magenta) prior to cell permeabilization. Cellular and bacterial DNA was stained with DAPI (cyan). Scale bar, 10 μm. Download FIG S6, PDF file, 0.2 MB. Copyright © 2021 Nozawa et al. 2021 Nozawa et al. This content is distributed under the terms of the Creative Commons Attribution 4.0 International license.

To further examine whether Nga inhibits transport from the ER to the Golgi apparatus, we performed the RUSH assay using SBP-EGFP-ManII, which is trafficked from the ER to the Golgi apparatus. Although EGFP-ManII was trafficked to the Golgi apparatus upon biotin treatment in control cells, EGFP-ManII colocalized with both the fragmented Golgi structures and the ER in GAS wild-type (WT)-infected cells even after biotin treatment (see Fig. S7). These results imply that ER-to-Golgi transport is affected by GAS infection.

10.1128/mBio.01974-20.7 FIG S7 Effect of GAS infection on the ER-Golgi transport. HeLa cells expressing streptavidin-KDEL and SBP-EGFP-ManII were treated with BFA or infected with GAS JRS4 for 2 h and incubated with biotin for 1 h. Cells were then fixed and immunostained for GM130. Cellular and bacterial DNA was stained with DAPI (cyan). Scale bars, 10 μm. Download FIG S7, PDF file, 0.2 MB. Copyright © 2021 Nozawa et al. 2021 Nozawa et al. This content is distributed under the terms of the Creative Commons Attribution 4.0 International license.

### Invading GAS and effector Nga disrupt epithelial integrity.

**FIG 5:**
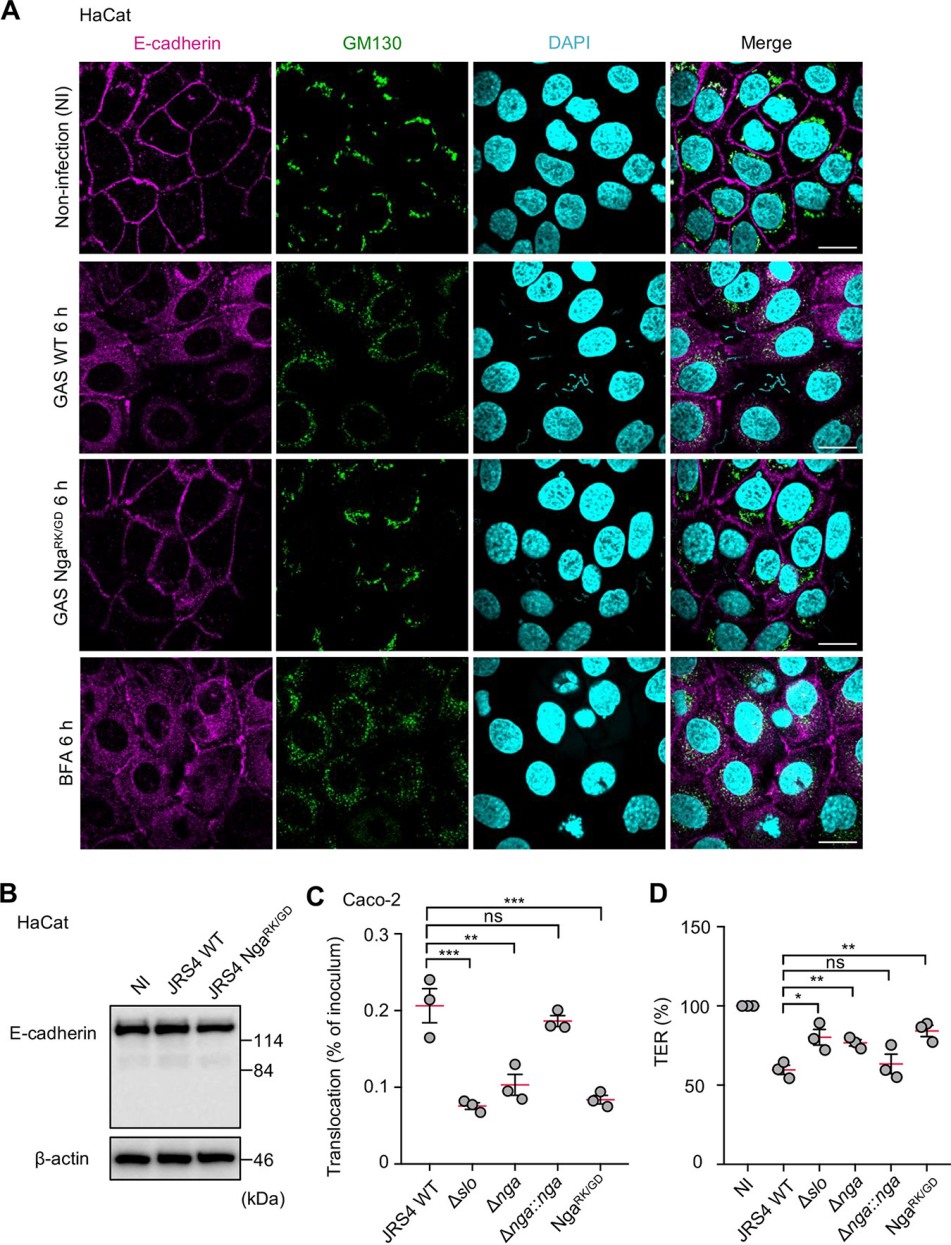
GAS affects E-cadherin trafficking and translocation of GAS through Nga. (A) HaCat cells infected with GAS strains were treated with BFA for 6 h, fixed, and immunostained with anti-E-cadherin (magenta) and GM130 (green). Cellular and bacterial DNA was stained with DAPI (cyan). Scale bars, 10 μm. (B) Western blot analysis of the indicated proteins in GAS-infected HaCat cells (6 h). (C) Caco-2 cells were grown on Millicell filters and then infected with GAS strains at an MOI of 10 for 6 h. Bacterial translocation was expressed as a percentage of GAS recovered from the medium beneath the monolayer at 6 h after infection. (D) Reduction of transepithelial electrical resistance (TER) in cells infected with GAS strains for 6 h. The TER value of noninfected cells was set at 100%. Data in panels C and D represent individual values (dots) and the means (magenta lines) ± SEMs from independent experiments. *P* values calculated by two-tailed Student’s *t* test. *, *P* < 0.05, **, *P* < 0.01, ***, *P* < 0.001; ns, not significant.

E-cadherin is critical for the cell-cell adhesion that holds epithelial cells tightly together; thus, E-cadherin is a crucial molecule for maintenance of the epithelial barrier. GAS can translocate across epithelial barriers by degrading junctional proteins, including E-cadherin (41, 42). Therefore, we examined whether the inhibition of anterograde trafficking by Nga affects E-cadherin localization and the ability of GAS to translocate across epithelial monolayers. Because the GAS protease, SpeB, degrades E-cadherin, we used a JRS4 strain that was defective in SpeB expression (43). Immunostaining of HaCat cells revealed that while E-cadherin was confined to the cell membrane in the noninfected cells and in cells infected with the strain Nga^R289K/G330D^, E-cadherin was present in substantial amounts in the cytoplasm in the JRS4-infected cells (Fig. 5A); this result suggests that the trafficking of endogenous E-cadherin to the cell membrane may be impaired by the Nga derived from the invading GAS. Furthermore, the HaCat cells treated with brefeldin A (BFA), which inhibits ARF and induces Golgi fragmentation (44), also exhibited E-cadherin redistribution similar to that induced by JRS4 infection (Fig. 5A). Because GAS induces calcium mobilization by SLO-induced pore formation and Nga-activity and calcium-binding cysteine protease, calpain, degrades E-cadherin (42, 45), we also examined the total E-cadherin level in HaCat cells. We found that JRS4 infection did not affect the cellular E-cadherin amounts (Fig. 5B). In addition, we showed that calpain inhibitor did not suppress Golgi fragmentation during infection (see Fig. S8). Collectively, these results suggest that Nga may not degrade E-cadherin but may alter the subcellular localization of E-cadherin during GAS infection in HaCat cells.

10.1128/mBio.01974-20.8 FIG S8 Effect of calpain inhibitor on the Golgi fragmentation. HeLa cells were treated with calpeptin at the indicated concentrations and infected with GAS JRS4 for 4 h. Cells were then fixed and immunostained for GM130. Cellular and bacterial DNA was stained with DAPI (cyan). Scale bars, 10 μm. The percentages of cells showing Golgi fragmentation were quantified. Data represent individual values (dots) (*n* > 100 cells per condition) and the means (magenta lines) ± SEMs from independent experiments. Download FIG S8, PDF file, 0.1 MB. Copyright © 2021 Nozawa et al. 2021 Nozawa et al. This content is distributed under the terms of the Creative Commons Attribution 4.0 International license.

We also examined the amount and localization of β-catenin in GAS-infected cells. β-catenin is involved in the cell-cell junction of epithelial cells. The total amount of β-catenin was not changed in GAS-infected cells (see Fig. S9A). In noninfected cells, β-catenin was localized to the cell membrane, but the signal of β-catenin in the cell membrane was decreased in JRS4 WT-infected cells (see Fig. S9B). In addition, this decreased cell membrane localization was not observed in Δ*slo*, Δ*nga*, and Nga^R289K/G330D^ GAS-infected cells (Fig. S9B). Therefore, it is suggested that GAS inhibits the transport of β-catenin to the cell membrane through SLO and Nga.

10.1128/mBio.01974-20.9 FIG S9 GAS inhibits the transport of β-catenin through SLO and Nga. (A) Total amount of β-catenin in GAS strain-infected cells. Western blot analysis of indicated proteins in GAS-infected HeLa cells (6 h). (B) HeLa cells were infected with GAS strains for 6 h, fixed, and immunostained with anti-β-catenin (green) and GM130 (magenta) antibodies. (C) Caco-2 cells were not infected or infected with JRS4 GAS for 4 h and immunostained for GM130. Cellular and bacterial DNA was stained with DAPI (cyan). Scale bars, 10 μm. Download FIG S9, PDF file, 0.4 MB. Copyright © 2021 Nozawa et al. 2021 Nozawa et al. This content is distributed under the terms of the Creative Commons Attribution 4.0 International license.

Redistribution of E-cadherin and β-catenin in the GAS-infected cells may increase bacterial translocation through the paracellular pathway. Because the HaCat, A549, and HeLa cells exhibit unstable junctional integrity, as indicated by their measured transepithelial electrical resistance (TER) (46), these epithelial cells are not suitable for assessing GAS translocation. Thus, for assaying GAS translocation, we selected polarized Caco-2 cells, which are widely used as an *in vitro* model of the epithelial barrier and have previously been used in experiments on GAS translocation (41, 42). We confirmed that GAS infection induced Golgi fragmentation even in Caco-2 monolayers (Fig. S9C). The apical surface of the Caco-2 monolayers was infected with either the JRS4 wild type or the JRS4 Δ*slo*, Δ*nga*, Δ*nga*::*nga*, or Nga^R289K/G330D^ mutant for 1 h, and the bacteriostatic agent, trimethoprim, was added to inhibit additional growth of extracellular and translocated bacteria. We confirmed that 25 μg/ml of trimethoprim bacteriostatically inhibited the additional growth of GAS strains (see Fig. S10). At 6 h postinfection, translocated bacteria were examined using the colony formation assay. Relative to that of the JRS4 wild type, the Δ*slo*, Δ*nga*, and Nga^R289K/G330D^ mutants exhibited markedly diminished translocation efficiency, whereas the Δ*nga*::*nga* mutant showed comparable translocation efficiency (Fig. 5C). In addition, the decrease of TER in Caco-2 cells was significantly suppressed in the Δ*slo*, Δ*nga*, and Nga^R289K/G330D^ GAS-infected cells compared to that in the JRS4 wild-type-infected cells (Fig. 5D). These results suggest that SLO and Nga facilitate GAS translocation across epithelial monolayers, perhaps by disrupting intracellular trafficking.

10.1128/mBio.01974-20.10 FIG S10 Trimethoprim bacteriostatically inhibits the growth of GAS. (A) Bacterial precultures were inoculated in THY containing different concentrations of trimethoprim. The cultures were incubated at 37°C for 4 h, and the OD_600_ was measured. (B) The bacterial culture of JRS4 (OD_600_ of 0.8) was adjusted to 1 × 10^7^ CFU/ml in Dulbecco’s modified Eagle’s medium (DMEM) with 25 μg/ml trimethoprim and incubated at 37°C for different time periods. The trimethoprim-treated bacteria for each time point were washed with PBS and plated on THY agar plates to determine the colony forming units. Download FIG S10, PDF file, 0.1 MB. Copyright © 2021 Nozawa et al. 2021 Nozawa et al. This content is distributed under the terms of the Creative Commons Attribution 4.0 International license.

### Invading GAS inhibits IL-8 secretion by using Nga.

**FIG 6:**
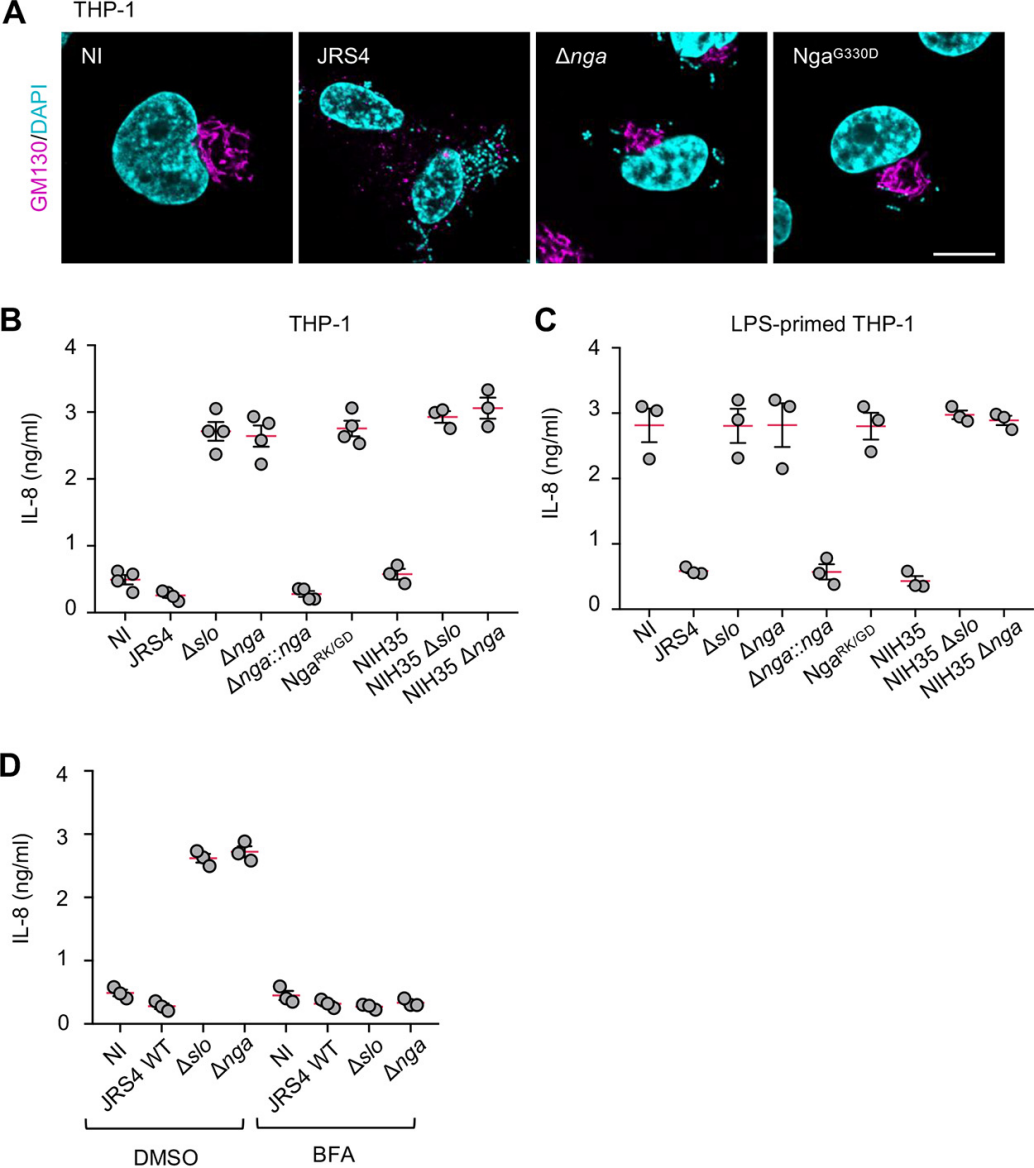
GAS inhibits IL-8 secretion process through SLO and Nga. (A) Differentiated THP-1 cells were infected with GAS JRS4 strains for 4 h, fixed, and immunostained with GM130 (magenta). Cellular and bacterial DNA was stained with DAPI (cyan). Scale bar, 10 μm. Nonprimed (B) or lipopolysaccharide (LPS)-primed (C) differentiated THP-1 cells were infected with GAS strains for 4 h. (D) The differentiated THP-1 cells were infected with GAS strains for 4 h with or without BFA. Supernatants were analyzed for the secretion of IL-8 by ELISA. Data in panels B, C, and D represent individual values (dots) and the means (magenta lines) ± SEMs from independent experiments.

GAS infection-induced Golgi fragmentation was also observed in differentiated THP-1 cells (Fig. 1A), and this fragmentation occurred through an Nga-dependent mechanism (Fig. 6A). Because macrophages produce the chemokine IL-8 in response to bacterial infection, we next determined whether invading GAS inhibited IL-8 secretion by using Nga. The differentiated THP-1 cells secreted IL-8 in response to infection by Δ*slo*, Δ*nga*, and Nga^R289K/G330D^ mutants but not JRS4 wild-type or Δ*nga*::*nga* strains (Fig. 6B), which suggests that SLO and Nga may inhibit IL-8 secretion by macrophages. Moreover, the invasive GAS strain, NIH35, blocked IL-8 secretion through an SLO- and Nga-dependent mechanism (Fig. 6B). To exclude the possibility that the lack of IL-8 production may be due to the suppression of IL-8 expression by SLO and Nga during GAS infection, we examined IL-8 secreted from the LPS-primed macrophages. The LPS-induced secretion of IL-8 was also inhibited by SLO and Nga (Fig. 6C), which indicates that the IL-8 secretion process was blocked by Nga during GAS infection. In addition, secretion of IL-8 in Δ*slo* and Δ*nga* GAS-infected cells was suppressed upon treatment with BFA (Fig. 6D), indicating that Golgi fragmentation results in the defect of IL-8 secretion during GAS infection. Taken together, it is suggested that invading GAS inhibits the IL-8 secretion pathway through SLO and Nga.

## DISCUSSION

**FIG 7:**
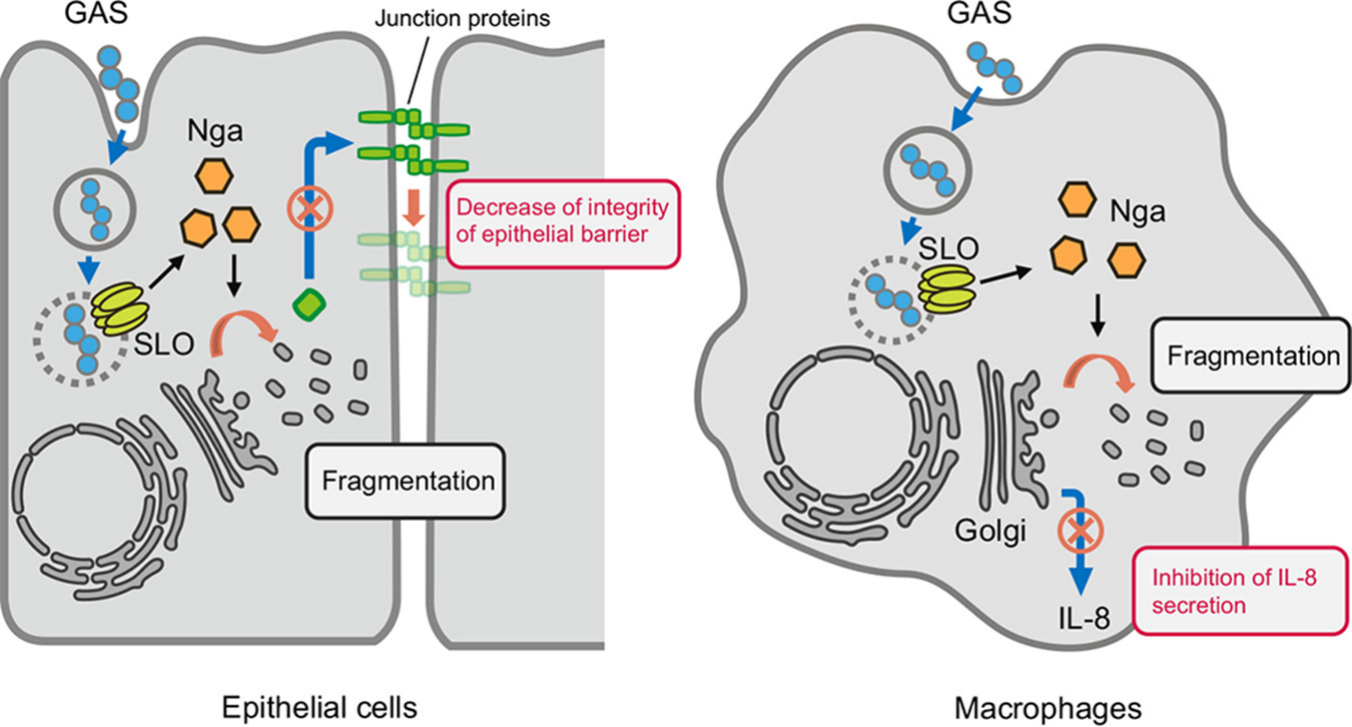
Invading GAS impairs host defense trafficking through SLO and Nga. In epithelial cells, invaded GAS secretes Nga into host cytosol through SLO and induces the Golgi fragmentation, which results in the impairment of maintenance of the cell-to-cell junction. In macrophages, IL-8 secretion in response to GAS infection is inhibited by Nga and SLO possibly through Golgi fragmentation.

Within bacterium-infected cells, highly complex interactions occur between the host immune system components and the bacterial pathogen, and unique molecular dynamics are frequently observed (25). We discovered in the present study that GAS invasion induced fragmentation of the Golgi complex and inhibited anterograde transport in an SLO- and Nga-dependent manner (Fig. 7). Notably, although GAS was found to translocate Nga into the host cytosol through an SLO-dependent mechanism without invading the host cell, a noninvasive GAS mutant (JRS4 Δ*fbp*) did not trigger Golgi fragmentation. These results uncover a previously unknown function of Nga that this effector protein performs in conjunction with other effectors and/or the GAS invasion process.

To our knowledge, this is the first report that GAS invasion disrupts the Golgi complex and the post-Golgi secretory pathway. The Golgi complex functions in sorting and trafficking in the central vacuolar system, and the Golgi apparatus and Golgi-associated trafficking have been widely reported to be affected by bacterial infection (25). For example, during *Shigella* infection, the *Shigella* effector protein IpaB induces cholesterol relocation and disrupts the Golgi complex and anterograde and retrograde transport (30); these modifications lead to the disruption of the host epithelial barrier and are associated with *Shigella* pathogenesis. We showed in the present study that GAS Nga activity also inhibits E-cadherin trafficking to the plasma membrane. E-cadherin promoted cell-to-cell adhesion and integrity of the epithelial barrier, and, accordingly, GAS translocation across epithelial monolayers was suppressed by the knockout of *slo* or *nga*. Sumitomo et al. reported that streptolysin S and a cysteine protease contribute to bacterial translocation perhaps by directly destabilizing intercellular junction proteins, such as E-cadherin (41, 42, 47). Our data suggest that invading GAS may support the translocation of extracellular GAS and may facilitate invasion into deeper tissues (Fig. 7).

Intriguingly, VirA from S. flexneri and EspG from enteropathogenic E. coli directly inactivate Rab1 and disrupt ER-to-Golgi trafficking in cells, and this disruption of the host secretory pathway results in the inhibition of IL-8 secretion from the infected cells (31); this suggests that the impairment of the post-Golgi secretory pathway may be linked to the attenuation of the inflammatory response. Lethal necrotizing fasciitis caused by GAS is characterized by the presence of few neutrophils at the infection site, and GAS expresses a secretory protein that degrades IL-8, which is crucial for neutrophil transmigration and activation (48–52). Thus, the absence of anterograde transport in GAS-invaded cells likely contributes to the GAS pathogenesis. Recently, newly emergent clade-associated strains of serotype M89 (M89 clade-3 strains) continue to be recognized as a cause of invasive diseases worldwide, and these strains were found to be genetically acapsular and, thus, incapable of producing the hyaluronic acid capsule (13, 53). Because the hyaluronic acid capsule is a critical virulence factor required for evading phagocytosis or endocytosis by host cells (54, 55), dissemination of these strains may be associated with the ability to invade host cells; however, the precise mechanism by which the acapsular characteristics influence the pathogenesis of these strains remains unknown. Although the expression of SLO and Nga is enhanced in M89 clade-3 strains, clade-associated and non-clade-associated M89 strains exhibit comparable intracellular survival (13, 53). Therefore, a previously unrecognized function of the Nga derived from intracellular GAS that suppresses host immune responses may be associated with the pathogenicity of the M89 clade-3 strains.

GAS invasion and the effector Nga are visibly linked to the morphological and functional destruction of the Golgi complex, but the molecular mechanism underlying this process has remained unclear. Unexpectedly, we found that the GAS JRS4 Δ*fbp*, which can inject Nga into the host cytosol, did not induce Golgi fragmentation; this suggests that Nga alone may be insufficient for inducing fragmentation. Although the proteins and/or events that function in Nga-dependent Golgi fragmentation during GAS invasion remain to be identified, the fragmentation was observed in all the GAS strains tested, which indicates that certain common characteristics shared among the strains are involved in producing this phenotype. Our time-lapse imaging analysis revealed that Golgi fragmentation occurred starting from 2 to 3 h postinfection, which coincides with the time of GAS invasion into the cytoplasm. We hypothesize that unidentified molecules secreted from GAS may be involved in the Golgi fragmentation. Notably, Nga has been reported to bind to SLO, and the interaction promotes their stability to enhance GAS virulence (56). Therefore, we cannot exclude the possibility that the conjugated Nga/SLO has particular functions after GAS endocytosis.

In summary, our findings indicate that GAS infection disrupts the Golgi-related network in host cells through the effector Nga and intracellular GAS, which then enables the translocation of GAS across epithelial barriers and the inhibition of IL-8 secretion by macrophages *in vitro*. Further investigation aimed at identifying other GAS molecules responsible for Golgi fragmentation will enhance our understanding of the pathogenicity of GAS.

## MATERIALS AND METHODS

### Bacterial strains and infection.

GAS strains JRS4, NIH35, and KUN-0014944 were grown in Todd-Hewitt broth supplemented with 0.2% yeast extract (THY) at 37°C. The isogenic mutant strains JRS4 Δ*slo*, JRS4 Δ*nga*, JRS4 Δ*nga*::*nga*, and JRS4 Nga^R289K/G330D^ have been described previously (57). JRS4 Δ*fbp*, JRS4 Δ*sagA*, NIH35 Δ*slo*, and NIH35 Δ*nga* were constructed using a two-step allele exchange by a method described previously. Overnight cultures were reinoculated in fresh THY and grown to the exponential phase (optical density at 600 nm [OD_600_] of 0.7 to 0.8), collected by centrifugation, and diluted with cell culture medium before use. Cell cultures in medium without antibiotics were infected for 1 h with GAS at an MOI of 100. Infected cells were washed with phosphate-buffered saline (PBS) and treated with 100 μg/ml gentamicin for an appropriate period to kill the bacteria that were not internalized.

### Cell culture.

HeLa, A549, and THP-1 cell lines were purchased from ATCC, the HaCat cell line was purchased from Cell Lines Service, HUVECs and NHEKs were purchased from PromoCell, and Caco-2 cells were purchased from the Riken Cell Bank. HeLa and A549 cells were maintained in Dulbecco’s modified Eagle’s medium (Nacalai Tesque) supplemented with 10% fetal bovine serum (FBS; Gibco) and 50 μg/ml gentamicin (Nacalai Tesque), and the THP-1 cells were cultured in RPMI 1640 medium (Nacalai Tesque) supplemented with 10% FBS and 50 μg/ml gentamicin. THP-1 cells were differentiated into macrophages by stimulating them with 50 ng/ml phorbol 12-myristate for 72 h. HUVECs were maintained with the endothelial cell growth medium 2 kit (PromoCell) supplemented with 10% FBS and 50 μg/ml gentamicin. NHEKs were cultured with the keratinocyte growth medium 2 kit (PromoCell), and Caco-2 cells were maintained in minimum essential medium (Wako) supplemented with 10% FBS and 50 μg/ml gentamicin. Cells were incubated in a 5% CO_2_ incubator at 37°C.

### Fluorescence microscopy.

Immunofluorescence analysis was performed using the following antibodies: anti-TOMM20 (1:100, ab78547; Abcam), anti-calnexin (1:100, 610524; BD Transduction Laboratories), anti-GM130 (1:100, 610822; BD Transduction Laboratories), anti-TGN46 (1:100, 13573-1-AP; Proteintech), anti-GFP (1:100) (GF200, 04363-24; Nacalai Tesque), anti-E-cadherin (1:100) (24E10, 3195; Cell Signaling Technology), anti-TOM20 (1:100) (F-10, sc-17764; Santa Cruz Biotechnology), and anti-β-catenin (1:100, ab16051; Abcam). The secondary antibodies used were anti-mouse or anti-rabbit IgG conjugated to Alexa Fluor 488 or 594 (numbers A32723, A32742, A32731, and A32740; Invitrogen). Cells were washed with PBS, fixed with 4% paraformaldehyde (PFA) in PBS (15 min), permeabilized with 0.1% Triton in PBS (10 min), washed with PBS, and blocked (room temperature, 1 h) with a skim milk solution (5% skim milk, 2.5% goat serum, 2.5% donkey serum, and 0.1% gelatin in PBS) or a bovine serum albumin (BSA) solution (2% BSA and 0.02% sodium azide in PBS). Next, the cells were probed (room temperature, 1 h) with the primary antibodies diluted in a blocking solution, washed with PBS, and labeled with the appropriate secondary antibody. To visualize bacterial and cellular DNA, cells were stained with 4′,6-diamidino-2-phenylindole (DAPI; Dojindo). Confocal fluorescence micrographs were acquired using an FV1000 laser scanning microscope (Olympus).

### Plasmids, transfection, and reagents.

Human FAPP1 cDNA was PCR amplified from the HeLa cell total mRNA and cloned into pcDNA-6.2/N-EmGFP-DEST (for N-terminal tagging) by using Gateway (Invitrogen) cloning technology as described previously (58). Ii-Str_SBP-EGFP-E-cadherin and Str-KDEL_ManII-SBP-EGFP were purchased from Addgene (plasmids 65288 and 65252). Polyethylenimine (Polysciences) and Lipofectamine 3000 (Invitrogen) were used for transfection. Z-VAD-FMK was purchased from Promega and was used at 50 μM. Cytochalasin D (cytD) was purchased from Tocris Bioscience and used at 5 μg/ml. Z-VAD-FMK or cytD was added 1 h before infection. Calpeptin was purchased from Tocris Bioscience and was used at 20 or 50 μM. Antibodies used for Western blot analysis were anti-E-cadherin (1:1,000) (24E10, 3195; Cell Signaling Technology), anti-β-actin (1:1,000) (13E5, 4970; Cell Signaling Technology), anti-β-catenin (1:100, ab16051; Abcam), and anti-glyceraldehyde-3-phosphate dehydrogenase (GAPDH) (1:1,000, H12, sc-166574).

### Measurement of NADase activity.

HeLa cells seeded at 1.5 × 10^5^ cells/well in 24-well plates were infected with the GAS strains for 3 h without killing of extracellular bacteria by gentamicin. Infected HeLa cells were scraped into chilled PBS, pooled with debris, and lysed in sterile water. The whole-cell lysates were cleared from the membrane fraction by centrifugation at 20,300 × *g* for 30 min at 4°C to obtain the cytosolic fraction, which was diluted 2-fold with 2× PBS. NAD^+^ (Nacalai Tesque) was added to the cytosolic fraction at 1 mM, and the mixtures were incubated at 37°C for 3 h. To develop reactions, NaOH (5 N) was added to the reaction mixtures, which were then incubated in the dark at room temperature for 30 min. Samples were analyzed by using a Wallac ARVO SX multilabel counter (PerkinElmer) at 340-nm excitation/460-nm emission to examine the fluorescence intensity of the remaining NAD^+^. NAD^+^ hydrolysis levels in lysates from the wild-type GAS-infected and noninfected cells were set to correspond to 100% and 0% NADase activity, respectively.

### RUSH assay.

To assess anterograde transport, the HeLa cells were transfected with the RUSH plasmid (Ii-Str_SBP-EGFP-E-cadherin, streptavidin-KDEL_ManII-SBP-EGFP), and at 24 h posttransfection, cells were infected with GAS strains as described above. After infection for 2 h, 40 μM biotin (Nacalai Tesque) was added to the cells, and after incubation for 1 h, the cells were fixed with 4% PFA (15 min) and immunostained with anti-GFP antibody without permeabilization. Images were acquired using confocal microscopy and analyzed using ImageJ software. Regions corresponding to the transfected cells were drawn, and the average intensity of anti-GFP staining in these regions was determined; >20 cells were analyzed under each condition, and three independent experiments were performed.

### Translocation assay.

Caco-2 cells were seeded at 2 × 10^5^ cells/well onto polycarbonate Millicell culture plate inserts (12-mm diameter, 3-μm pore size; Millipore) and cultured for 5 days. To determine the Caco-2-cell monolayer integrity, the TER of the monolayers on the filter was measured using a Millicell-ERS device (Millipore), and monolayers with measured TER values of 450 to 500 Ω/cm^2^ were used in experiments. After the polarized monolayers were infected for 1 h with GAS (MOI = 100), the nonadherent bacteria were removed by washing the upper chamber with PBS, and the medium was switched to a medium containing 25 μg/ml trimethoprim to inhibit the additional growth of GAS. The ability of GAS to translocate across monolayers was assessed through quantitative culturing of the medium. Each medium sample obtained from the lower chamber at 6 h postinfection was serially diluted and plated on THY agar plates to determine CFU values.

### Chemokine and cytokine secretion.

THP-1 cells were seeded at 2 × 10^5^ cells/well in 24-well plates and differentiated for 72 h. After infection with GAS for 4 h, the supernatant was collected and centrifuged, and the IL-8 released into the supernatant was quantified by using a human IL-8 enzyme-linked immunosorbent assay (ELISA) kit (Proteintech) according to the manufacturer’s instructions.

### Statistical analysis.

Values, including plotted values, represent means ± standard errors of the means (SEMs). Data were tested using the two-tailed Student’s *t* test, and a *P* value of <0.05 was considered significant. GraphPad Prism 8 was used for statistical analyses.
