# Supplementary figures and images for: Intracellular Group A Streptococcus Induces Golgi Fragmentation To Impair Host Defenses through Streptolysin O and NAD-Glycohydrolase
Source: mBio. 2021 Feb 9;12(1):e01974-20. doi: 10.1128/mBio.01974-20 (PMC7885101; doi:10.1128/mBio.01974-20)

Supplementary Figure 1

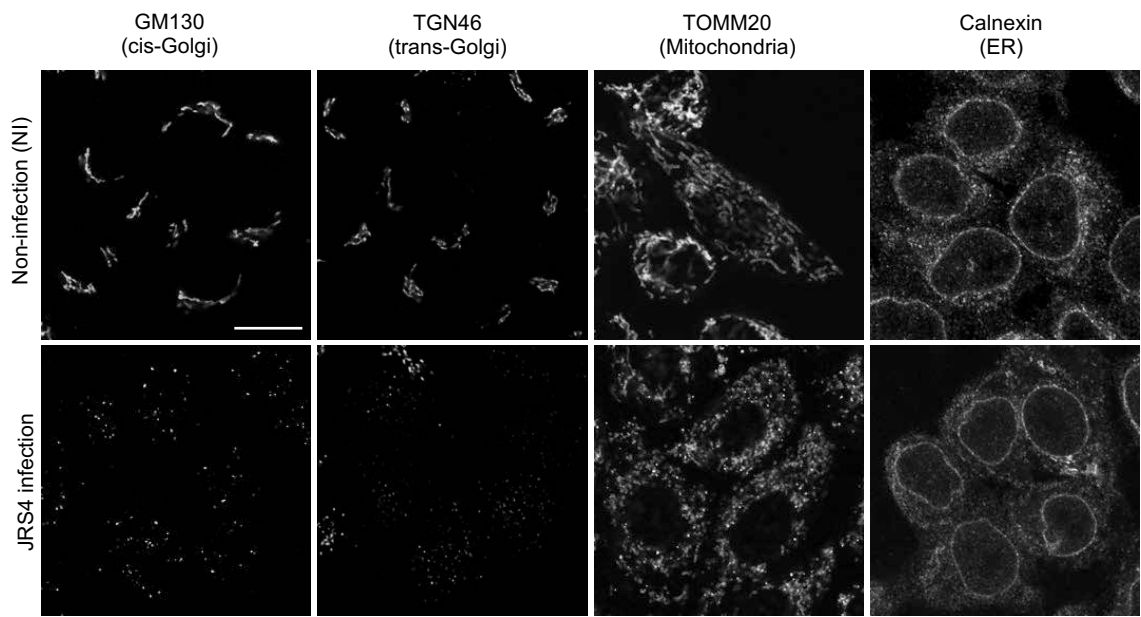

Supplement: FIG S1 [file mBio.01974-20-sf001.pdf]

Supplementary Figure 2

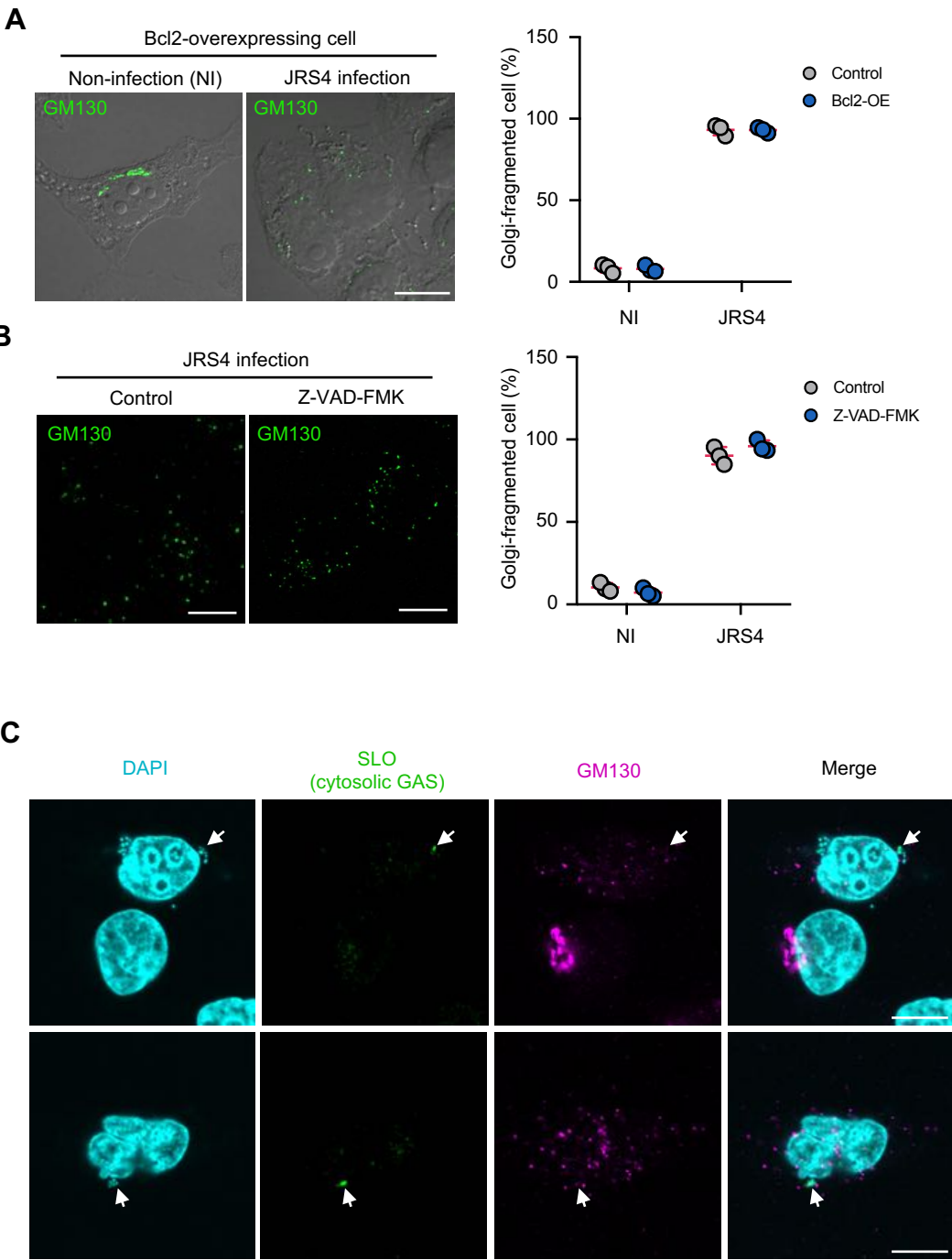

Supplement: FIG S2 [file mBio.01974-20-sf002.pdf]

Supplementary Figure 3

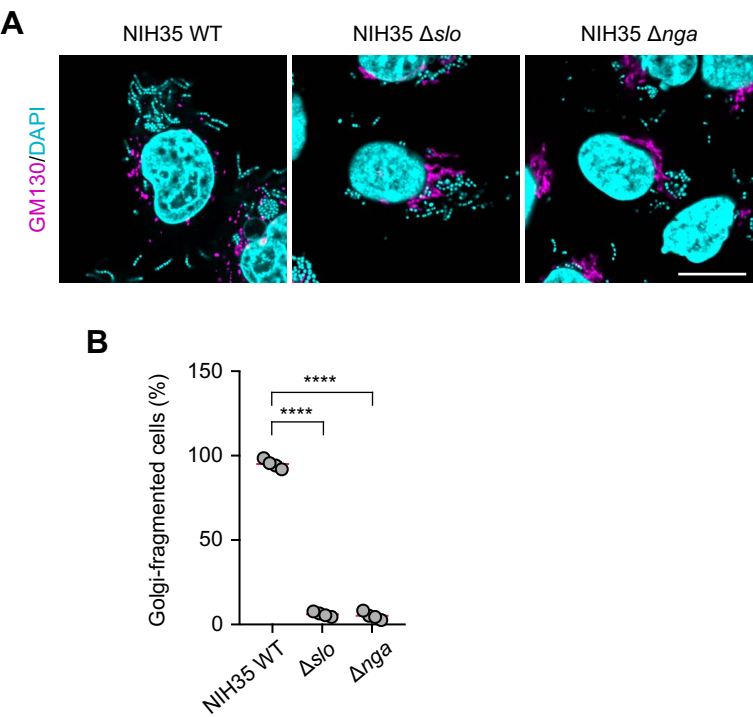

Supplement: FIG S3 [file mBio.01974-20-sf003.pdf]

Supplementary Figure 4

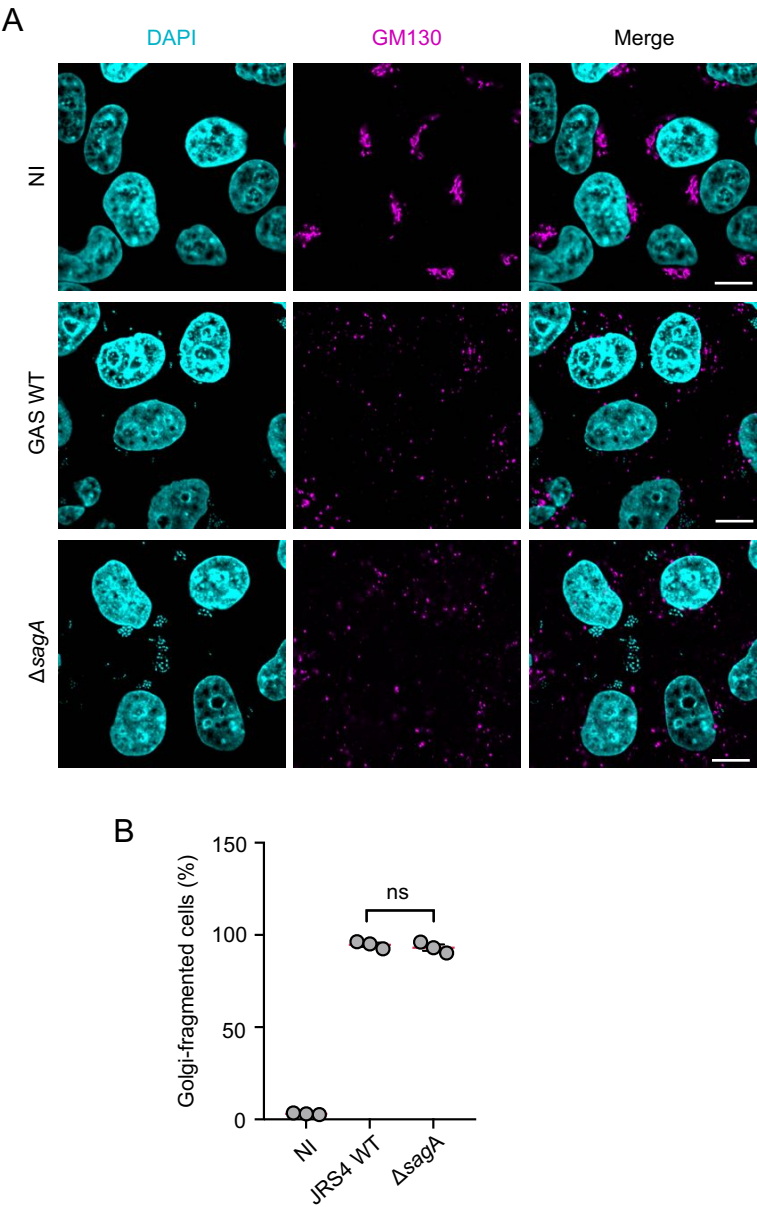

Supplement: FIG S4 [file mBio.01974-20-sf004.pdf]

Supplementary Figure 5

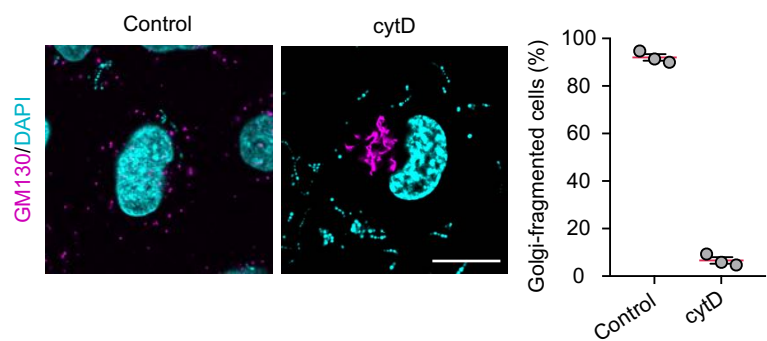

Supplement: FIG S5 [file mBio.01974-20-sf005.pdf]

Supplementary Figure 6

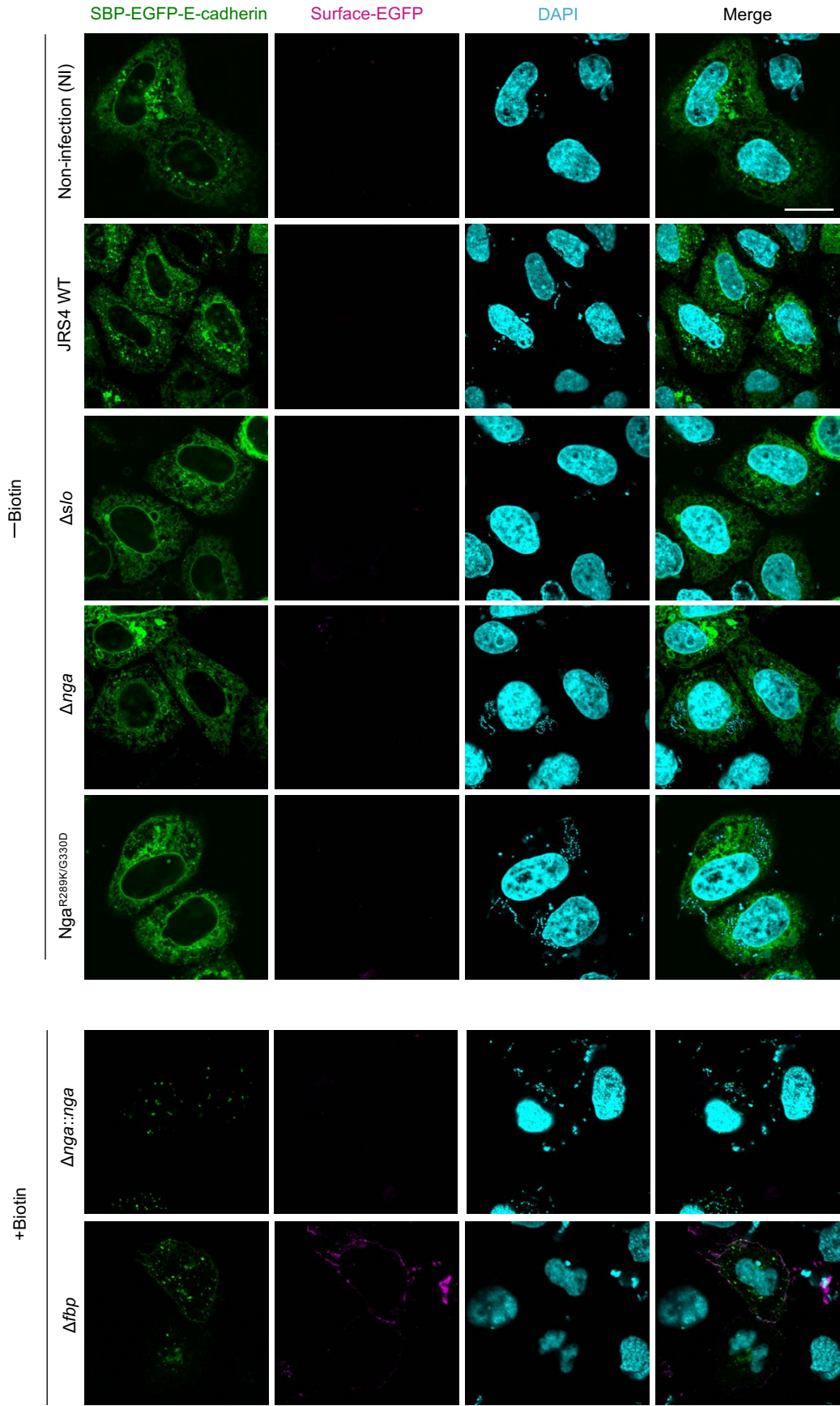

Supplement: FIG S6 [file mBio.01974-20-sf006.pdf]

Supplementary Figure 7

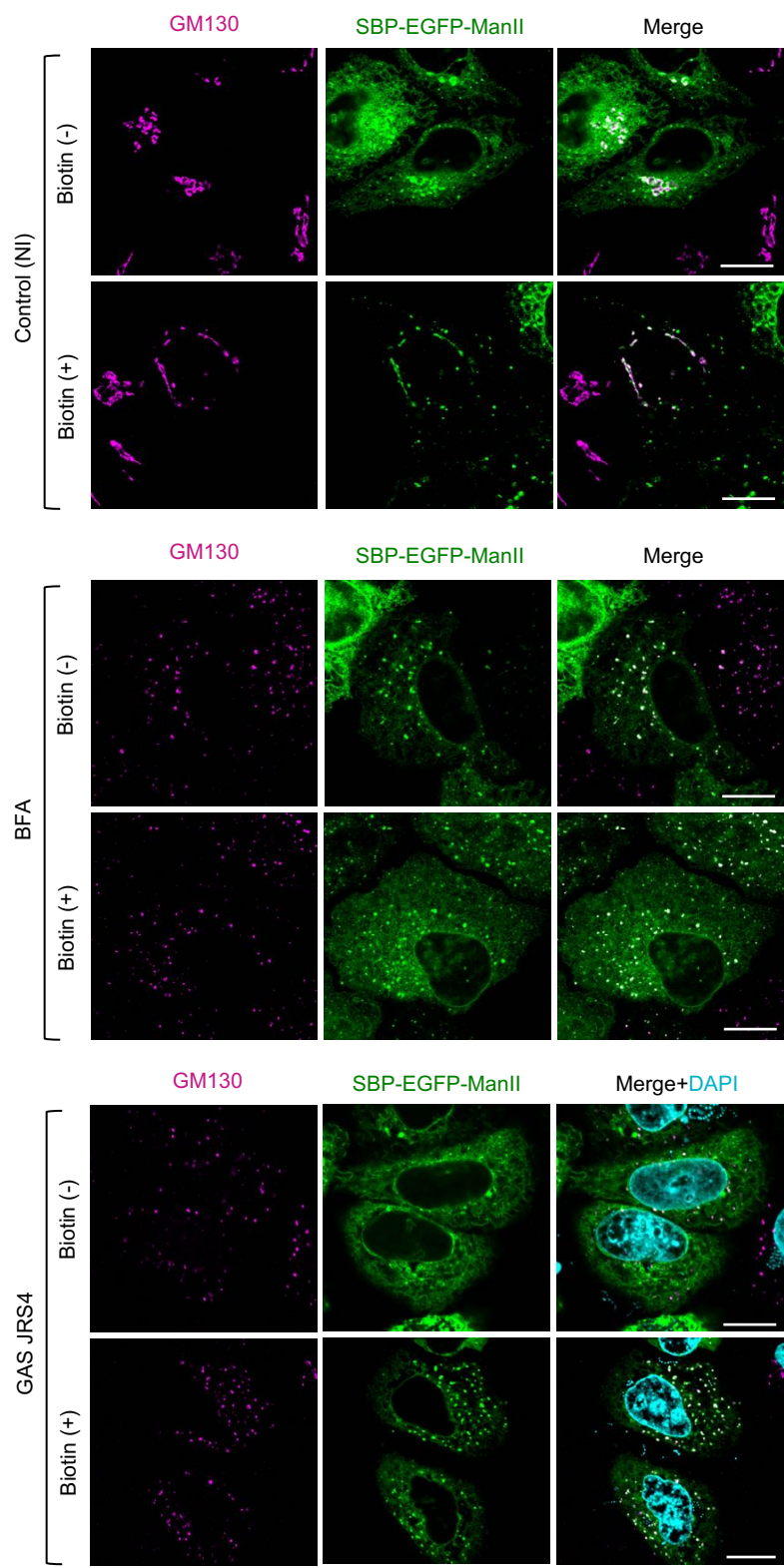

Supplement: FIG S7 [file mBio.01974-20-sf007.pdf]

Supplementary Figure 8

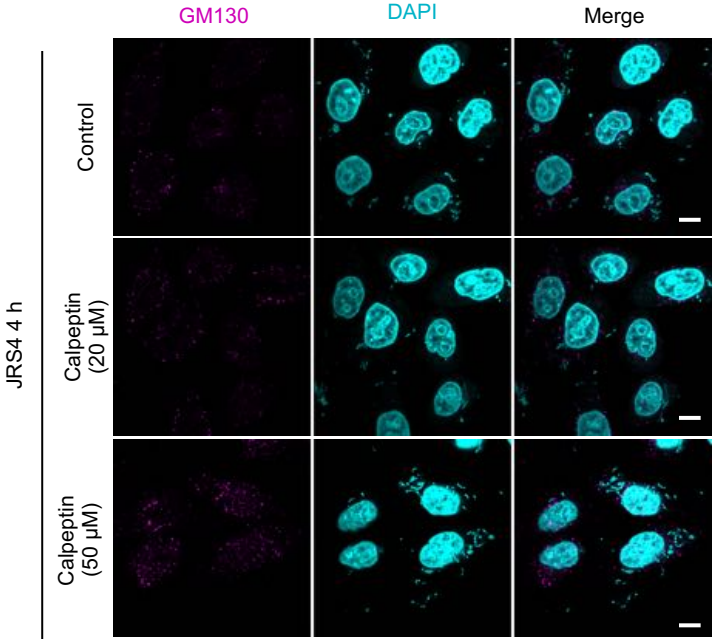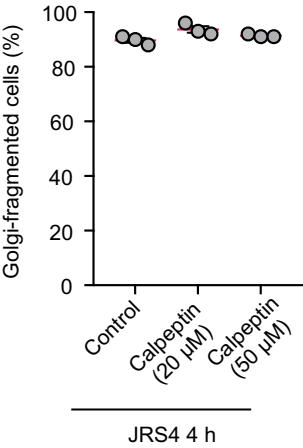

Supplement: FIG S8 [file mBio.01974-20-sf008.pdf]

# Supplementary Figure 9

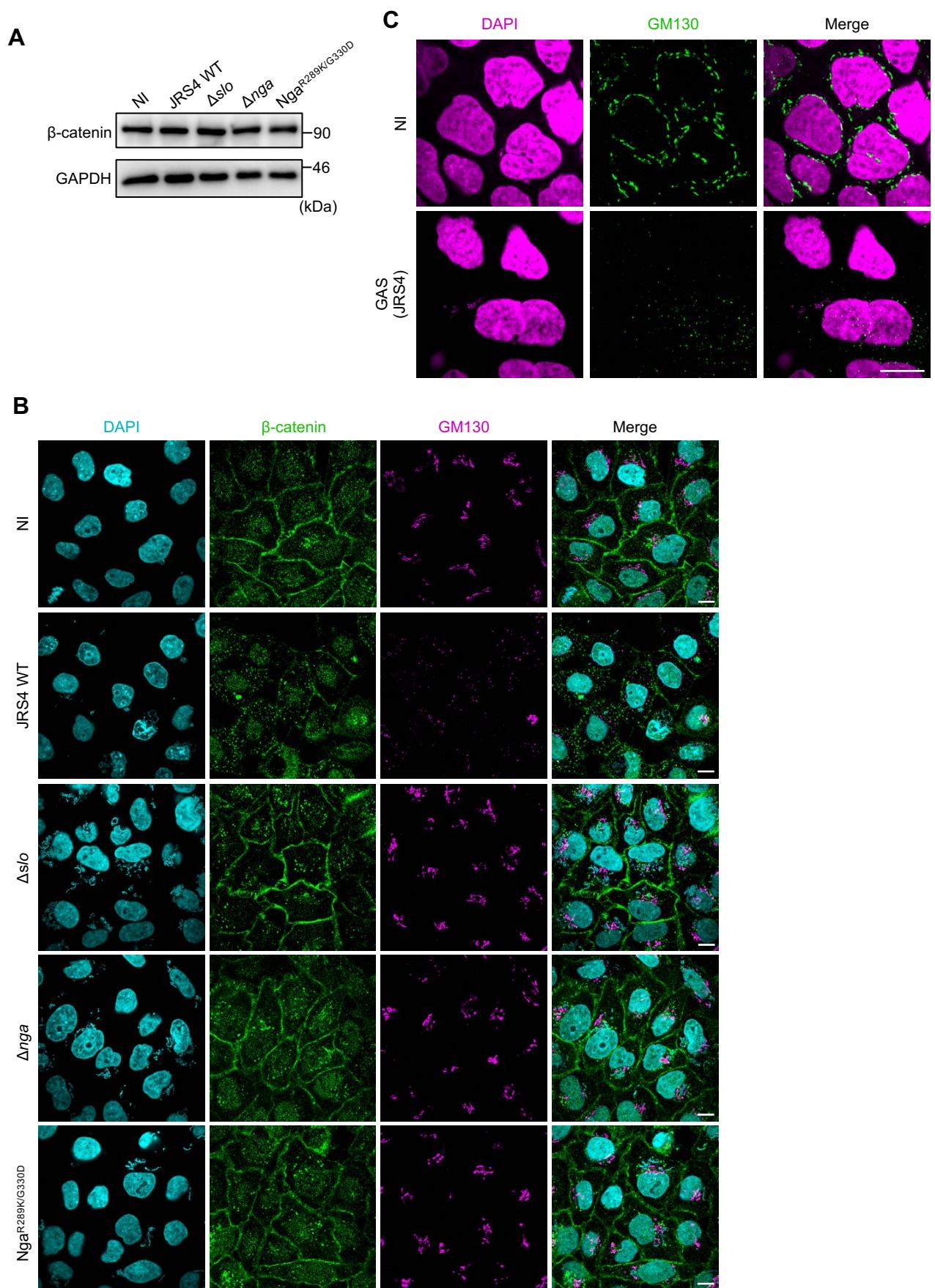

Supplement: FIG S9 [file mBio.01974-20-sf009.pdf]

Supplementary Figure 10

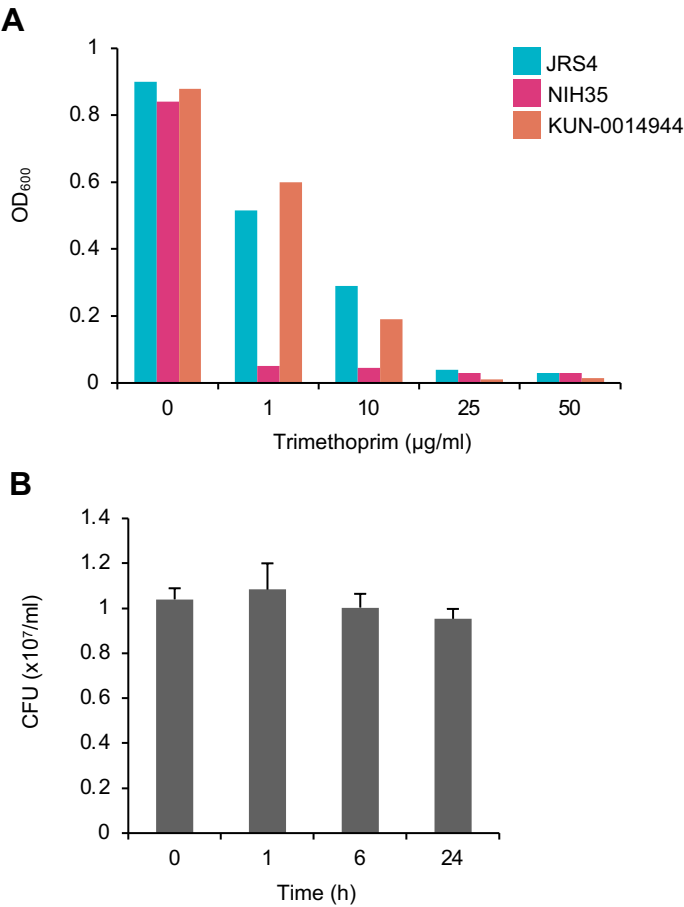

Supplement: FIG S10 [file mBio.01974-20-sf010.pdf]
